# Nano- and micro-structural control of WO_3_ photoelectrode films through aqueous synthesis of WO_3_·H_2_O and (NH_4_)_0.33_WO_3_ precursors†

**DOI:** 10.1039/d0ra01321h

**Published:** 2020-03-20

**Authors:** Hiroaki Uchiyama, Yuki Nagayasu

**Affiliations:** Department of Chemistry and Materials Engineering, Kansai University 3-3-35 Yamate-cho Suita 564-8680 Japan h_uchi@kansai-u.ac.jp +81-6-6368-1121 ext. 6131; Kansai University Japan

## Abstract

Nano- and micro-structured tungsten trioxide (WO_3_) photoelectrode films were prepared through an aqueous solution route. WO_3_ precursor layers were deposited on glass substrates through heterogeneous nucleation from (NH_4_)_10_W_12_O_41_ aqueous solutions at 50–60 °C. The crystal phase of the precursors changed from WO_3_·H_2_O to (NH_4_)_0.33_WO_3_ with increasing (NH_4_)_10_W_12_O_41_ concentration (*x*), which involved a morphological change from micron-scale plates to nano-scale fine particles. The WO_3_·H_2_O and (NH_4_)_0.33_WO_3_ layers were thermally converted to the monoclinic WO_3_ phase. The fine-particle WO_3_ films obtained from (NH_4_)_0.33_WO_3_ layers showed a better photoanodic performance in the UV range below 350 nm, which was attributed to the larger surface area arising from the porous structure. On the other hand, platy-particle WO_3_ films were obtained from WO_3_·H_2_O layers, which exhibited strong light scattering in the visible range, and resulted in an enhanced photoanodic response at wavelengths above 375 nm.

## Abstract 2

Nano- and micro-structured WO_3_ films obtained from WO_3_·H_2_O and (NH_4_)_0.33_WO_3_ precursors prepared by an aqueous route showed a good photoanodic performance depending on their morphologies.

## Graphical abstract

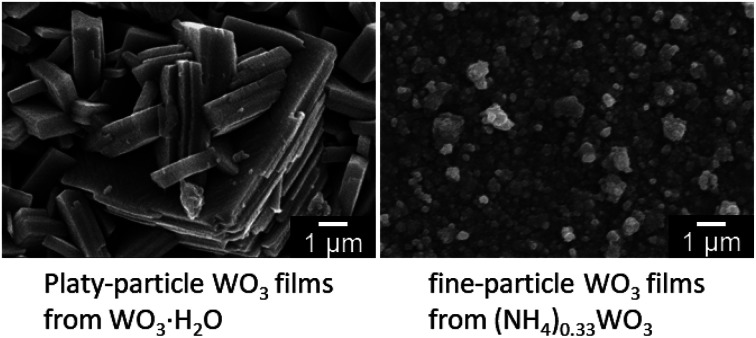

## Introduction

Tungsten oxide (WO_3_) film materials have been receiving attention as visible-light-responsive photoanode materials owing to their relatively small band gap (2.5–2.8 eV) and photoelectrochemical stability in aqueous solutions.^1–5^ In a photoelectrochemical system, the nano- and micro-structures of electrode materials significantly affect the device performance. Nanostructures (such as nanoparticles, nanowires, and nanorods) provide a larger surface area, that is, a larger number of active sites for photoelectrochemical reactions, which results in a more effective photo-energy conversion.^2,6–8^ In addition, submicron- and micron-scale porous structures are reported to work as light-scattering layers, where the path length of the incident light in the film materials increases, which leads to more effective light absorption.^9–13^ Thus, the fabrication techniques of nano- and micro-structures are widely investigated for the practical application of WO_3_ materials.

The solution route is an effective synthetic method for nano- and micro-structured inorganic materials, where the size, morphology, crystal phase, and crystallinity of the products are influenced by processing parameters such as the concentration and temperatures of the precursor solutions. Many works have attempted to prepare novel WO_3_ materials using hydrothermal and solvothermal approaches.^5,7,14–18^ Hong *et al.* reported that WO_3_ nanocrystals could be synthesized by a hydrothermal reaction followed by additional calcination, and the products showed good photocatalytic and photoelectrochemical activities.^14^ Zhang *et al.* prepared WO_3_ nanotree films by hydrothermal oxidation of W substrates, which exhibited a high coloration efficiency as electrochromic materials.^16^ Zheng *et al.* synthesized thin films consisting of WO_3_ nanoplates with an exposed (002) plane, where orthorhombic WO_3_·H_2_O nanoplates were prepared as precursors by a hydrothermal method and then converted to monoclinic WO_3_ by calcination.^17^ Wang *et al.* suggested a 2-step hydrothermal method for making WO_3_ nanoplate array films with (002) oriented facets on fluorine-doped tin oxide (FTO) glass substrates, where the WO_3_ films exhibited a high electrochemical performance for water splitting.^5^ Liu *et al.* prepared a WO_3_–CuS nanosheet heterojunction with enhanced photocatalytic performance by a simple on-step solvothermal method.^18^ These studies suggest that solution routes are promising for the fabrication of nanostructured WO_3_ materials with enhanced device properties.

Tungsten species are reported to exist as monomeric tungstate ions (WO_4_^2−^) or paratungstate ions (HW_6_O_21_^5−^, H_2_W_12_O_42_^10−^, and so forth) in an aqueous media,^19–23^ and such tungstate ions can deposit as various types of hydrous tungsten oxides (WO_3_·*x*H_2_O) and tungstates (H_2_WO_4_, H_4_WO_5_ and so forth).^6,8,24,25^ Previously, we have reported the preparation of nanostructured WO_3_ particle materials, where WO_3_·H_2_O layered platy particles were first obtained as precursors through an aqueous solution process and then thermally converted to monoclinic WO_3_.^26^ In this case, the macroscopic layered structures of the precursors remained even after the thermal conversion to WO_3_. Such synthetic routes through morphology-controlled precursors have also been reported by several groups.^6,17,24^ A variation in the chemical composition and crystal phase of WO_3_ precursors would allow us to control the nano- and micro-structures of the resultant WO_3_ materials.

In this work, we address the nano- and micro-structural control of WO_3_ film materials through the control of the crystal phase of the precursor tungsten species by the aqueous solution route. Here, the reaction temperature for the nucleation and growth of tungsten species was set to 50–60 °C, which is milder than hydrothermal techniques, because such mild conditions are considered to be preferable for the phase control of WO_3_ precursors containing metastable phases. First, WO_3_ precursor layers were deposited on glass substrates through heterogeneous nucleation from (NH_4_)_10_W_12_O_41_ aqueous solutions under mild conditions below 60 °C, and then thermally converted to monoclinic WO_3_ materials. Moreover, we evaluate the photoelectrochemical properties of the WO_3_ heat-treated films, and investigate the effect of the nano- and micro-structures on the photoanodic performances.

## Experimental

HCl aqueous solutions at pH 1.0 were prepared by diluting approximately 36.0 mass% hydrochloric acid (Wako Pure Chemical Industries, Osaka, Japan) with purified water. (NH_4_)_10_W_12_O_41_·5H_2_O (0.010–0.50 g, Wako Pure Chemical Industries) was added and dissolved in 20 cm^3^ of the HCl solutions under stirring at 80 °C for 3 min ([(NH_4_)_10_W_12_O_41_·5H_2_O] (*x*) = 0.17–8.5 mM). Soda-lime glass (20 mm × 40 mm × 1.0 mm), silica glass (20 mm × 40 mm × 1.0 mm), and fluorine-doped tin oxide (FTO) glass substrates (20 mm × 40 mm × 1.0 mm) were dipped in the (NH_4_)_10_W_12_O_41_ solutions, and then the solutions were aged at 50 or 60 °C for 1–3 days. After the aging, precursor layers were deposited on glass substrates. The precursor layers were washed with purified water and dried at 60 °C for 1 day. The WO_3_ precursor films thus obtained were heated at 600 °C for 1 day in air for the thermal conversion to WO_3_, where heat treatment was performed at a heating rate of 5 °C min^−1^.

The crystalline phases of the precursor and heat-treated films were identified by X-ray diffraction (XRD) measurements in the ordinary 2*θ*/*θ* mode using an X-ray diffractometer (Model Rint 2550V, Rigaku, Tokyo, Japan) with CuKα radiation operated at 40 kV and 300 mA. The morphologies of the film samples were observed using field emission scanning electron microscopy (FE-SEM) (Model JSM-6500F, JEOL, Tokyo, Japan). Optical transmission spectra were measured on the samples using an optical spectrometer (V-570, JASCO, Tokyo, Japan), where a FTO glass substrate was used as the reference.

The photoanodic properties of the WO_3_ films were evaluated in a three-electrode cell using a potentiostat (HZ-7000, Hokuto Denko, Osaka, Japan) consisting of the film electrode sample, a platinized Pt electrode, and a saturated calomel electrode (SCE) as the working, counter, and reference electrodes, respectively, and of a buffer solution of pH 7, which was an aqueous solution of 0.2 M Na_2_B_4_O_7_, 0.14 M H_2_SO_4_, and 0.3 M Na_2_SO_4_, as the supporting electrolyte.

Action spectra of the films were measured at 1.0 V *versus* SCE, where a xenon lamp light was monochromatized using a monochromator (SPG-100s, Shimadzu, Kyoto, Japan). The intensity of the monochromatized light was measured using a power meter (NOVA, PD300-UV, Ophir Japan, Saitama, Japan), and was approximately 23 μW at a wavelength of 500 nm. For this measurement, the film was first illuminated for 10 s, and then the light was turned off. The difference in current before and after turning off the light was taken as the photocurrent. Quantum efficiency, that is, the incident photon-to-current efficiency (IPCE), was calculated from the photocurrent and incident light intensity.

## Results and discussion

### Preparation of WO_3_ precursor films

**Fig. 1:**
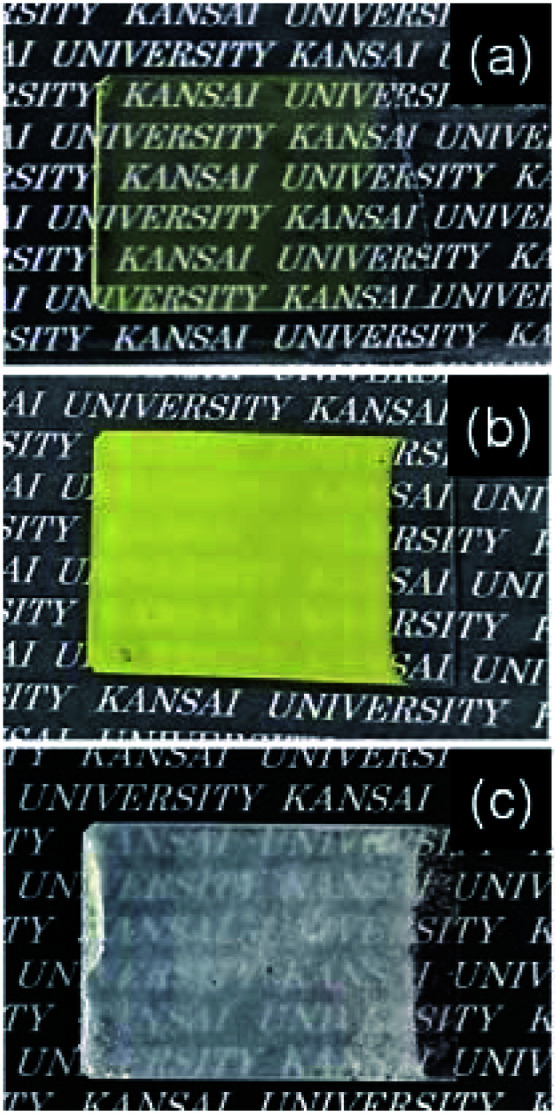
Optical micrographs of the WO_3_ precursor layers on soda-lime glass substrates prepared at *x* = 0.17 (a), 1.7 (b), and 8.5 (c) mM.

WO_3_ precursor layers were deposited on soda-lime glass substrates from the (NH_4_)_10_W_12_O_41_ solutions of [(NH_4_)_10_W_12_O_41_·5H_2_O] (*x*) = 0.17–8.5 mM by aging at 60 °C for 1 day. Fig. 1 shows the appearance of the WO_3_ precursor layers on the soda-lime glass substrates. Yellowish layers formed on the substrates at *x* = 0.17–1.7 mM, where the amount of the precursors increased with increasing *x* (Fig. 1a and b). The color of the precursor layers changed to white at a higher *x* above 4.3 mM (Fig. 1c), where partial cracking was often observed on the surface.

**Fig. 2:**
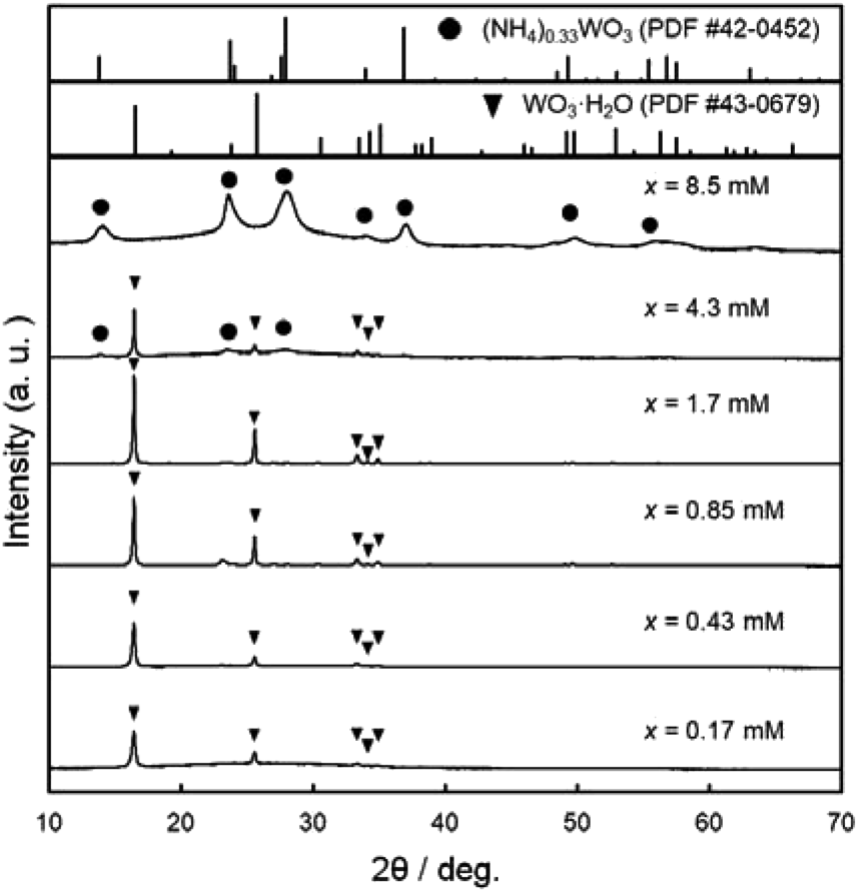
XRD patterns of the WO_3_ precursor layers on soda-lime glass substrates prepared at *x* = 0.17–8.5 mM.

Fig. 2 shows the XRD patterns of the WO_3_ precursor layers prepared at *x* = 0.17–8.5 mM. The yellowish precursors obtained below *x* = 1.7 mM were identified as the WO_3_·H_2_O phase. On the other hand, the increase in the (NH_4_)_10_W_12_O_41_·5H_2_O concentration (*i.e.*, the increase in the amount of NH_4_^+^ ions in the solutions) resulted in the formation of the ammonium tungstate phase. The diffraction peaks of the (NH_4_)_0.33_WO_3_ phase appeared over *x* = 4.3 mM, and single-phase (NH_4_)_0.33_WO_3_ products were obtained at *x* = 8.5 mM. The paratungstate ion, W_12_O_42_^10−^, changes to monomeric tungstate ions (*e.g.*, WO_4_^2−^ and HWO_4_^−^) or other paratungstate species (*e.g.*, HW_6_O_21_^5−^ and H_2_W_12_O_42_^10−^) in aqueous solutions depending on the pH value, concentrations, and coexisting ions in the solutions.^19–23^ Here, W_12_O_42_^10−^ ions were thought to change to WO_4_^2−^ ions under acidic conditions, and then deposit as the WO_3_·H_2_O phase at lower NH_4_^+^ contents and as the (NH_4_)_0.33_WO_3_ phase at higher NH_4_^+^ contents.

**Fig. 3:**
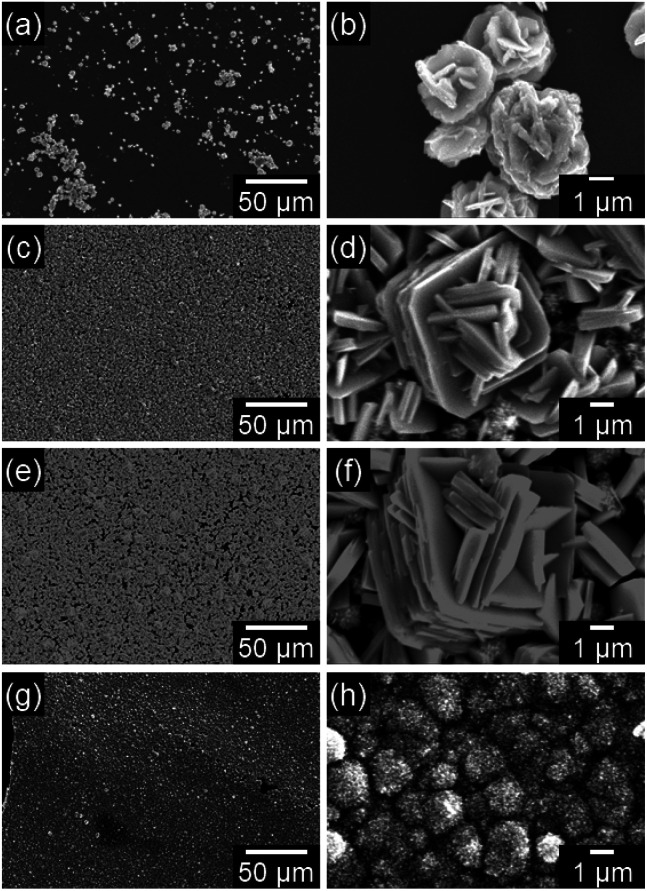
SEM images of the WO_3_ precursor layers on soda-lime glass substrates prepared at *x* = 0.17 (a and b), 0.85 (c and d), 1.7 (e and f), and 8.5 (g and h) mM.

Fig. 3 shows the SEM images of the WO_3_ precursor layers prepared at *x* = 0.17–8.5 mM by aging at 60 °C for 1 day. The morphologies of the precursors drastically varied with the changing crystal phase. Aggregates of platy particles were observed for the yellowish WO_3_·H_2_O layers obtained at *x* = 0.17–1.7 mM (Fig. 3a–f), where the number of platy particles on the substrates increased with increasing *x* (Fig. 3a, c and e). Moreover, the size of platy units became larger from approximately 1 μm to 7–10 μm with increasing *x* from 0.17 to 1.7 mM (Fig. 3b, d and f). The white (NH_4_)_0.33_WO_3_ layers prepared at *x* = 8.5 mM were found to consist of fine particles below 50 nm in size (Fig. 3g and h).

### Preparation WO_3_ heat-treated films and their photoanodic properties

We attempted to make WO_3_ photoelectrode films from WO_3_·H_2_O layers consisting of platy particles (*x* = 1.7 mM) and (NH_4_)_0.33_WO_3_ layers consisting of fine particles (*x* = 8.5 mM). In the previous section, the precursor layers were obtained by aging at 60 °C for 1 day, where the WO_3_·H_2_O and (NH_4_)_0.33_WO_3_ products each had problems concerning the exposure of the glass substrates, which was undesirable for the electrochemical evaluations. In the case of the WO_3_·H_2_O layers of *x* = 1.7 mM, the amount of precursor was not sufficient to cover the glass substrates, and thus the substrate was partially exposed (Fig. 3a, c and e). The substrate exposure was solved by increasing the aging time to 3 days (*x* = 1.7 mM, aged at 60 °C) (ESI, Fig. S1a and b†). On the other hand, the (NH_4_)_0.33_WO_3_ layers of *x* = 8.5 mM were often cracked and delaminated owing to the larger amounts of precursors. Low-temperature aging at 50 °C (*x* = 8.5 mM, aged for 1 day) reduced the deposition amount of the (NH_4_)_0.33_WO_3_ precursor, which resulted in the suppression of cracking (ESI, Fig. S1c and d†). These WO_3_·H_2_O and (NH_4_)_0.33_WO_3_ layers with no substrate exposure were used to make the WO_3_ heat-treated films.

**Fig. 4:**
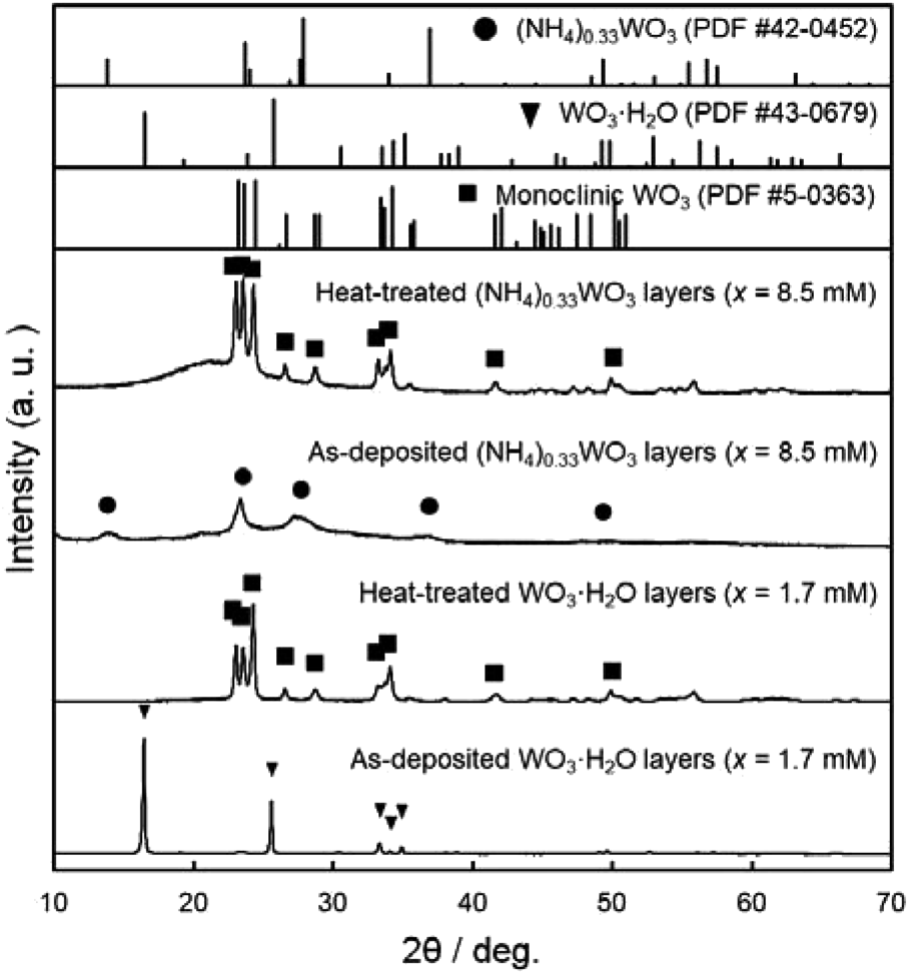
XRD patterns of the WO_3_ precursor layers and heat-treated films prepared on silica glass substrates at *x* = 0.17 mM (aged at 60 °C for 3 days) and 8.5 mM (aged at 50 °C for 1 day).

WO_3_ heat-treated films were prepared from the WO_3_·H_2_O layers consisting of platy particles (*x* = 1.7 mM, aged at 60 °C for 3 days) and the (NH_4_)_0.33_WO_3_ layers consisting of fine particles (*x* = 8.5 mM, aged at 50 °C for 1 day). The precursors were deposited on silica glass substrates, and then thermally converted to WO_3_ films by heating at 600 °C for 1 day. Fig. 4 shows the XRD patterns of the WO_3_ precursors and heat-treated films on silica glass substrates. The diffraction patterns attributed to the monoclinic WO_3_ phase were detected for the heat-treated products.

**Fig. 5:**
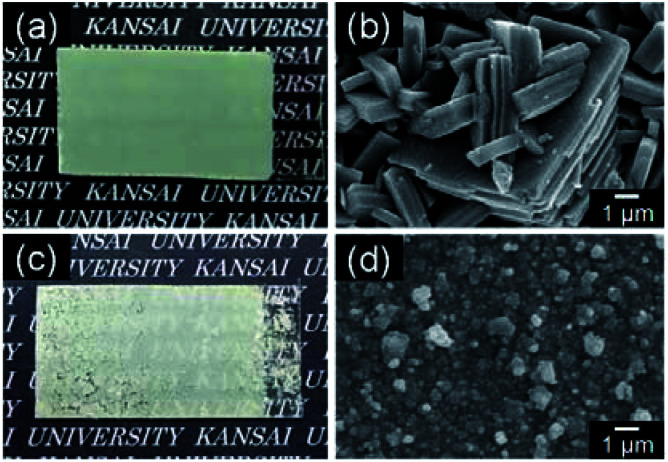
Optical micrographs (a and c) and SEM images (b and d) of the WO_3_ heat-treated films obtained from WO_3_·H_2_O (a and b) and (NH_4_)_0.33_WO_3_ (c and d) precursor layers on silica glass substrates.

**Fig. 6:**
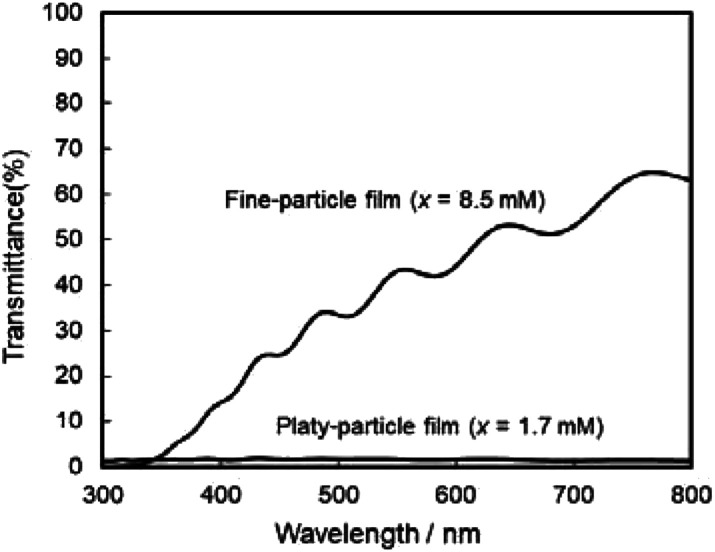
Cross-section SEM images of the WO_3_ heat-treated films obtained from WO_3_·H_2_O (a) and (NH_4_)_0.33_WO_3_ (b) precursor layers on silica glass substrates.

Fig. 5 shows the micrographs and SEM images of the WO_3_ heat-treated films on silica glass substrates. The heat-treated films were cloudy and the film color became light yellow after the heat treatment (Fig. 5a and c). Crack-free films were obtained from the WO_3_·H_2_O layers (Fig. 5a), while cracking and delamination were occasionally observed for the (NH_4_)_0.33_WO_3_ layers after heating (Fig. 5c). The nano- and micro-structures of the precursors remained even after the thermal conversion to the monoclinic WO_3_ phase (Fig. 5b and d). Platy particles of approximately 10 μm were observed for the WO_3_ heat-treated films prepared from the WO_3_·H_2_O layers (Fig. 5b). Previously, we have reported the topotactic transformation of WO_3_·H_2_O plates to WO_3_ platy particles by heating, where the flat face of WO_3_ plates was the (001) plane of monoclinic WO_3_.^26^ Thus, in the present work, the flat face of the platy particles observed on the substrates was deduced to be the (001) plane of monoclinic WO_3_, as well as the previous one. On the other hand, porous films consisting of fine particles were obtained from the (NH_4_)_0.33_WO_3_ layers (Fig. 5d). The cross-section SEM images of the WO_3_ heat-treated films are shown in Fig. 6. The thickness of the heat-treated films obtained from the WO_3_·H_2_O and (NH_4_)_0.33_WO_3_ layers was approximately 4.3 (Fig. 6a) and approximately 2.6 μm (Fig. 6b), respectively.

**Fig. 7:**
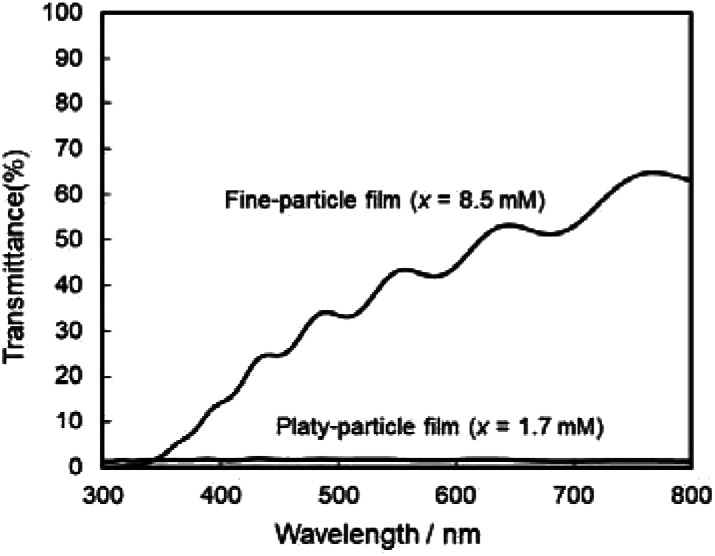
Optical transmission spectra of the WO_3_ heat-treated films obtained from WO_3_·H_2_O and (NH_4_)_0.33_WO_3_ precursor layers on FTO glass substrates.

**Fig. 8:**
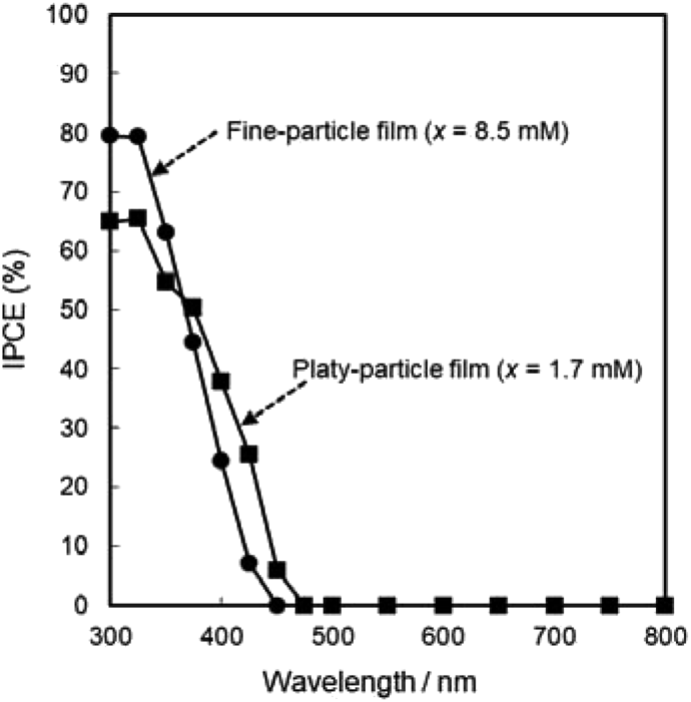
Action spectra of the WO_3_ heat-treated films obtained from WO_3_·H_2_O and (NH_4_)_0.33_WO_3_ precursor layers on FTO glass substrates.

The photoanodic properties were evaluated for the WO_3_ heat-treated films obtained from WO_3_·H_2_O (platy particles) and (NH_4_)_0.33_WO_3_ layers (fine particles), where the heat-treated films were prepared on FTO glass substrates. We checked that the change of substrates from silica glass to FTO glass didn't affect the crystal phases and morphologies of the products. Fig. 7 shows the optical transmission spectra of the WO_3_ heat-treated films on FTO glass substrates. As shown in Fig. 5, the WO_3_ heat-treated films were cloudy (Fig. 5a and c), which was attributed to the light scattering by the submicron- and micron-scale particles and aggregates in the film layers. From the optical transmittance analysis, the heat-treated films actually exhibited a low transparency in the visible range of 300–800 nm, even though WO_3_ materials absorb UV and visible light at wavelengths below 470 nm. Especially, a very low transmittance (almost 0%) was detected in the entire visible range for the platy-particle WO_3_ films obtained from WO_3_·H_2_O layers. On the other hand, in the case of the fine-particle WO_3_ films obtained from (NH_4_)_0.33_WO_3_ layers, the transmittance slightly increased with increasing wavelength from 350 nm. The low transparency of the platy-particle films was thought to result from the enhanced light scattering owing to the larger micron-scale platy particles. Fig. 8 shows the action spectra of the WO_3_ heat-treated films, which were measured at a working electrode potential of 1.0 V *versus* SCE. The photocurrent under monochromatized light was detected at wavelengths below 450 nm, which agreed with the photoabsorption of WO_3_ materials (band gap energy of WO_3_ is 2.5–2.8 eV). In the UV range below 350 nm, the IPCE value of the fine-particle films was higher than that of the platy-particle films. Because the photoelectrochemical reactions occur on the surface of the electrode materials, the larger surface area, arising from the porous structure consisting of fine particles, would result in a more efficient energy conversion. On the other hand, at wavelengths over 375 nm, the platy-particle films exhibited higher IPCE than the fine-particle films. As shown in Fig. 7, the platy-particle films showed strong light scattering in the visible range. Such light scattering has been reported to elongate the light path length in film layers, which enhances the light utilization efficiency.^9–13^ Here, the micron-scale platy particles obtained from WO_3_·H_2_O layers could act as a light-scattering layer, which results in the more effective photo-energy conversion in the visible range.

## Conclusions

We achieved the morphological control of WO_3_ film materials by an aqueous solution process. WO_3_·H_2_O and (NH_4_)_0.33_WO_3_ layers were prepared on glass substrates through heterogeneous nucleation from (NH_4_)_10_W_12_O_41_ aqueous solutions, and then thermally converted to monoclinic WO_3_ films. The variation in the crystal phase of the precursors led to the morphological change of the resultant WO_3_ films. Micron-scale platy-particle films were obtained from WO_3_·H_2_O layers, while (NH_4_)_0.33_WO_3_ layers provided nano-scale fine-particle films. The larger surface area of the fine-particle films resulted in a better photoanodic response in the UV range below 350 nm. On the other hand, the micron-scale platy particles exhibited strong light scattering in the visible range, which elongated the light path length in the film layers, leading to the improvement of the light utilization efficiency at wavelengths over 375 nm. We propose that the morphological control techniques of WO_3_ film materials through the aqueous solution route and the photoelectrochemical properties depending on the nano- and micro-structures are useful for the development of high-efficiency solar cell devices.

## Conflicts of interest

There are no conflicts to declare.

## Supplementary Material

RA-010-D0RA01321H-s001
