# Correction: Nano- and micro-structural control of WO_3_ photoelectrode films through aqueous synthesis of WO_3_·H_2_O and (NH_4_)_0.33_WO_3_ precursors

**DOI:** 10.1039/d0ra90033h

**Published:** 2020-04-09

**Authors:** Hiroaki Uchiyama, Yuki Nagayasu

**Affiliations:** Department of Chemistry and Materials Engineering, Kansai University 3-3-35 Yamate-cho Suita 564-8680 Japan h_uchi@kansai-u.ac.jp +81-6-6368-1121 ext. 6131; Kansai University Japan

## Abstract

Correction for ‘Nano- and micro-structural control of WO_3_ photoelectrode films through aqueous synthesis of WO_3_·H_2_O and (NH_4_)_0.33_WO_3_ precursors’ by Hiroaki Uchiyama *et al.*, *RSC Adv.*, 2020, **10**, 11444–11449.

The Royal Society of Chemistry regrets that an incorrect version of Fig. 6 was included in the original article. The correct version of Fig. 6 is presented below.

**Fig. 6:**
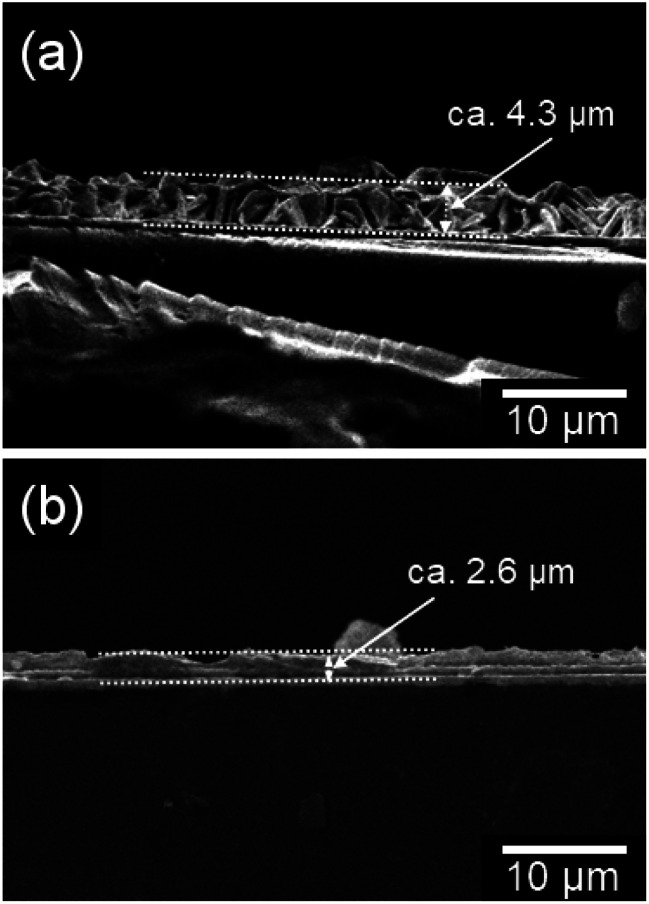
Cross-section SEM images of the WO_3_ heat-treated films obtained from WO_3_·H_2_O (a) and (NH_4_)_0.33_WO_3_ (b) precursor layers on silica glass substrates.

The Royal Society of Chemistry apologises for these errors and any consequent inconvenience to authors and readers.

## Supplementary Material

