# Supplementary material for: Nano- and micro-structural control of WO3 photoelectrode films through aqueous synthesis of WO3·H2O and (NH4)0.33WO3 precursors
Source: RSC Adv. 2020 Mar 20;10(19):11444–9. doi: 10.1039/d0ra01321h (PMC9050517; doi:10.1039/d0ra01321h)
Supplement: RA-010-D0RA01321H-s001 [file RA-010-D0RA01321H-s001.pdf]

Electronic Supplementary Material (ESI) for RSC Advances.
This journal is © The Royal Society of Chemistry 2020

Nano- and micro-structural control of WO_3 photoelectrode films through aqueous synthesis of $WO_3 \cdot H_2O$ and $(NH_4)_{0.33}WO_3$ precursors

Hiroaki Uchiyama* and Yuki Nagayasu

E-mail address: h_uchi@kansai-u.ac.jp (H. Uchiyama).

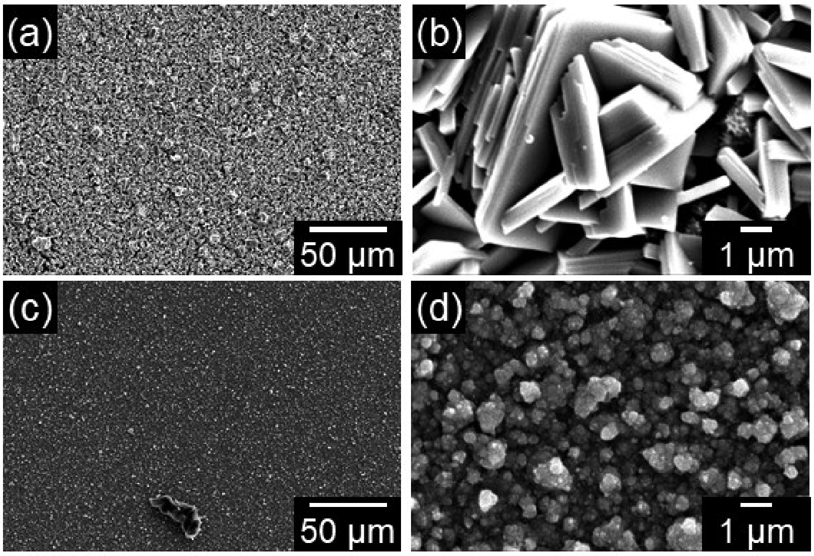

Fig. S1 SEM images of the WO_3 precursor layers prepared on silica glass substrates at x = 0.17 mM (aging at 60 °C for 3 days) (a–b) and 8.5 mM (aging at 50 °C for 1 day) (c–d).
